# Long-term treatment patterns and outcomes in IgG4-related disease – a retrospective single-center cohort study focusing on rituximab

**DOI:** 10.1007/s00296-025-06065-1

**Published:** 2026-01-13

**Authors:** Ariane Hammitzsch, Noemi Ferraro, Quirin Bachmann, Uwe Heemann, Philipp Moog

**Affiliations:** Rheumatology, Department of Nephrology, TUM School of Medicine and Health, TUM University Hospital, Technical University of Munich, Ismaninger Strasse 22, 81675 Munich, Germany

**Keywords:** IgG4-RD, Rituximab, B cell depleting therapy, Therapeutic outcome, Relapse, Safety aspects, Cohort study, Retrospective study, Observational study

## Abstract

IgG4-related disease (IgG4-RD) is a rare immune-mediated, fibroinflammatory disease with heterogenous presentations and no standardized treatment recommendations. This study investigates long-term efficacy and safety of current therapeutic strategies with a focus on rituximab. A retrospective analysis was conducted on 24 patients diagnosed with IgG4-RD at a German tertiary center (2010–2020) using the 2020 revised comprehensive diagnostic criteria. Patients were recruited from Rheumatology and Nephrology. Data included organ involvement, laboratory results, histology, treatments, relapses, therapy-related damage, and comorbidities. The cohort included 12 males and 12 females, median age at diagnosis 53 (95%CI 37.0; 61.0) years. Males had more affected organs (2.4 vs. 1.6; p = 0.036) and higher IgG4 levels (>5× upper limit: 33.3% vs. 16.7%; p = 0.036). Immunosuppressive therapy was initiated in 87.5% of patients, with glucocorticoids (GC) universally included. Rituximab was administered to 71.4%, mainly as a 4 x 375 mg/m² regimen (77.3%), with a median follow-up post first rituximab of 51.0 months (95%CI 27.0; 63.0). Adverse events were not more frequent with rituximab compared to other regimens. At last visit, 47.6% were off immunosuppressives and 38.1% remained on GC. Active organ involvement declined, though 16.7% showed organ damage progression. Relapses were frequent (81.0%), but less common upon rituximab initiation (26.7%). This representative IgG4-RD cohort demonstrates that long-term treatment with rituximab as maintenance therapy is generally effective and safe regardless of therapeutic regimen. Despite this glucocorticoid use remains high, highlighting the need for guidelines to standardize the use of glucocorticoids and DMARDs like rituximab in both induction and maintenance therapy.

**Supplementary Information:**

The online version contains supplementary material available at 10.1007/s00296-025-06065-1.

## Introduction

IgG4-related disease (IgG4-RD) is a rare immune-mediated fibro-inflammatory condition first identified in 2003 [1]. It can affect single or multiple organs and often mimics infections, malignancies, or other rheumatic diseases, posing diagnostic challenges [2]. Early recognition and treatment are critical to prevent irreversible organ damage or unnecessary interventions [3]. Key histopathological features include dense lymphoplasmacytic infiltrates rich in IgG4-positive plasma cells, storiform fibrosis, and obliterative phlebitis. Elevated serum IgG4 levels occur in about 80% of patients, and rapid clinical improvement with glucocorticoids (GC) or B cell depletion therapy is typical [4, 5].

Four major clinical subgroups of IgG4-RD have been identified: pancreato-hepatobiliary, retroperitoneal fibrosis/aortitis, head and neck limited, and Mikulicz syndrome with systemic manifestations [5]. These subgroups vary by ethnicity, sex, and serum IgG4 serum levels [6]. There is also a distinction between predominantly proliferative and fibrotic disease phenotypes, with yet unknown clinical significance [7].

Most patients respond well to GC, but relapse rates are high, with 46% of patients relapsing within two years after tapering or stopping GC [8]. Maintenance therapies with GC-sparing agents like methotrexate, azathioprine or mycophenolate mofetil have variable success [9]. Rituximab, a CD20-targeting antibody, is effective for induction and maintenance and is considered first-line after GC in most countries. However, data on its long-term efficacy (beyond 24 months) and safety in IgG4-RD are limited. The optimal dosing intervals and regimens required to maintain safe remission also remain unclear. Recently, the anti-CD19 antibody inebilizumab was approved for induction treatment in the United States due to reduced flare risk in a Phase III trial [10]. Despite growing evidence, official guidelines for immunosuppressive management beyond induction are lacking.

This study retrospectively examines the clinical course, treatments, and outcomes of 24 IgG4-RD patients at a German tertiary center, focusing on therapeutic strategies and disease progression, with particular attention to long-term maintenance with rituximab.

## Materials and methods

### Study population

This retrospective observational study was approved by the Institutional Review Board of the Technical University of Munich on 17.10.2016 (204/16 S) and followed the STROBE recommendations for cohort studies by the EQUATOR Network (See Supplementary material) [11]. All adult patients diagnosed with IgG4-RD in the Department of Nephrology (including Rheumatology) between 2010 and 2020 were identified and screened. Inclusion criteria (> 18 years of age, at least one follow-up, classifiable within the 2020 RCD [revised comprehensive diagnostic] criteria) and exclusion criteria (< 18 years of age, conflicting diagnosis with regard to the exclusion criteria of the 2019 ACR/EULAR classification criteria, not classifiable within the 2020 RCD criteria, no follow-up) were applied [4, 12]. Figure S1 shows a flow chart of the study concept. The study was performed in accordance with the Declaration of Helsinki (Version Dec. 2024) and patients were included after confirmed diagnosis and written informed consent.

### Data collection

Clinical and demographic data were collected retrospectively between September and December 2020 from medical records of routine visits, covering the period from diagnosis to last follow-up. Relapse and treatment response were determined based on clinical, serological, or imaging criteria (PET/CT, PET/MRI, or contrast-enhanced ultrasonography). Relapse was further characterized by the necessity to intensify immunosuppressive therapy (including GC and DMARDs), while response was defined by the ability to taper immunosuppression.

### Statistical analysis

Patient characteristics were described overall and by sex, relapse versus non-relapse, and rituximab versus other immunosuppressive therapy. Non-normally distributed continuous variables are presented as medians with 95% Confidence Interval (95%CI) and compared using the Mann-Whitney test. Categorical variables are shown as counts and percentages and analyzed with Fisher’s exact test. Overall Time-to-relapse endpoint was estimated by the Kaplan–Meier method. P-values < 0.05 were considered significant throughout the study. Analyses were performed using GraphPad Prism version 10.4.1 (GraphPad Software, Boston, USA).

## Results

### Baseline clinical characteristics

Twenty-six patients with IgG4-related disease (IgG4-RD) were initially screened. Two were excluded due to conflicting diagnosis (eGPA) or not meeting the 2020 revised comprehensive diagnostic criteria (2020 RCD), leaving 24 adult patients for analysis with a median follow-up of 54.5 months (95%CI 34.0–65.0) as presented in Table 1. Half were male, and median age at diagnosis was 52 years (95%CI 37.0–61.0), with no significant sex difference. Median time to diagnosis was 10.5 months (95%CI 2.0–25.0). According to the 2020 RCD criteria, 50% had a definite, 41.6% a probable, and 8.3% a possible diagnosis [12]. Probable diagnoses were more frequent in females (66.7% vs. 16.7%, *p* = 0.036). Allergies were present in 20.8%, with no sex difference. All patients had biopsies; over 90.5% had > 10 IgG4 + plasma cells per high power field. Storiform fibrosis was evident in 62.5%, while obliterative phlebitis was rare (12.5%), with no sex-related histologic differences.

**Fig. 1:**
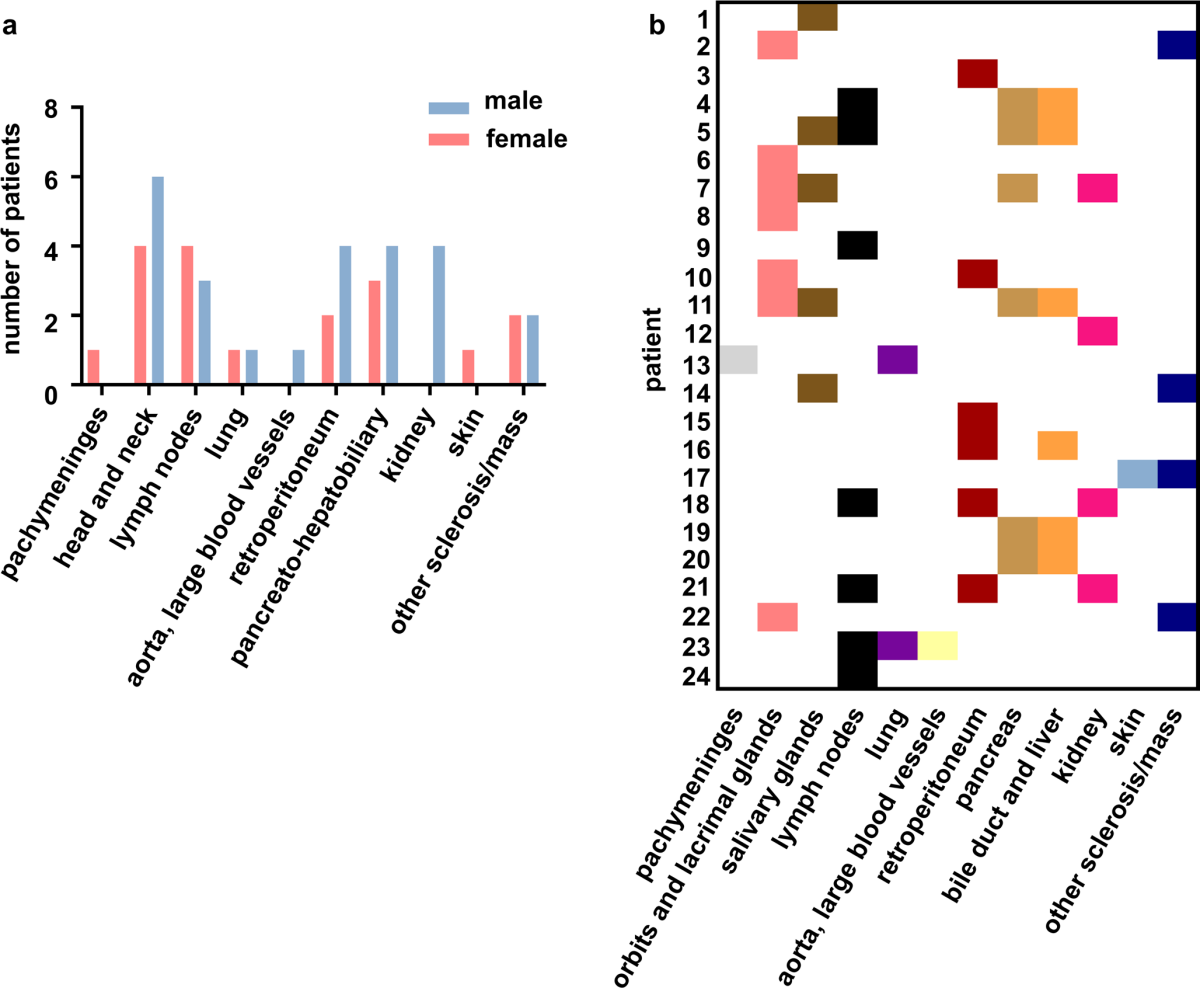
Summary of affected organs and patterns of organ involvement at baseline. **a** No significant differences in the involved organs at baseline between male and female patients. **b** Heterogenous pattern of organ involvement at baseline

Sixteen patients (66.7%) had multi-organ involvement (defined as ≥ 2 organs), with single-organ disease being more common in females (6/12 females vs. 2/12 males, ns). Mean number of affected organs was significantly higher in males (2.4, SD 1.0, 95%CI 1.6; 3.0) than females (1.6, SD 1.0, 95% CI 1.0; 2.3; *p* = 0.039). The most affected sites were head and neck (41.7%), lymph nodes, and pancreato-hepatobiliary area (each 29.2%), without sex differences (Fig. 1a and Table S1). Kidney involvement occurred only in males (4/12 patients, ns). Organ patterns were heterogeneous and representative of prior cohort data (Fig. 1b).

**Table 1:** Baseline demographic characteristics overall and by sex

	All (*n* = 24)	Male (*n* = 12)	Female (*n* = 12)	*p* value
Age at diagnosis (median, 95%CI)	52.0 (37.0; 61.0)	53.0 (21.0; 67.0)	52.0 (32.0; 61.0)	0.989
Time to diagnosis (months; median, 95%CI)	10.5 (2.0; 25.0)	10.5 (1.0; 122.0)	8.5 (2.0; 28.0)	0.831
Duration of follow-up (months; median, 95%CI)	54.5 (34.0; 65.0)	54.5 (31.0; 65.0)	54.5 (19.0; 68.0)	0.943
Male *n* (%)	12 (50.0)	–	–	–
Race				
Caucasian	22 (91.7)	11 (91.7)	11 (91.7)	ns
Asian	2 (8.3)	1 (8.3)	1 (8.3)	ns
IgG4 concentration (mg/dL, median, 95%CI)	361.5 (106.0; 844.0)	473.0 (242.0; 3030.0)	112 (52.6; 491.0)	0.045
Biopsy performed *n* (%)	24 (100.0)	12 (100.0)	12 (100.0)	ns
Obliterative phlebitis *n* (%)^#^	3 (12.5)	1 (8.3)	2 (16.7)	> 0.999
Storiform fibrosis *n* (%)^#^	15 (62.5)	7 (58.3)	8 (66.7)	> 0.999
> 10 IgG4 + cells/HPF *n* (%)^∞^	19 (90.5)	9 (90.0)	10 (90.9)	> 0.999
2020 revised comprehensive diagnostic criteria *n* (%)				
Definite	12 (50.0)	8 (66.7)	4 (33.3)	0.220
Probable	10 (41.7)	2 (16.7)	8 (66.7)	0.036
Possible	2 (8.3)	2 (16.7)	0 (0.0)	0.478
Number of organs affected at baseline (mean, SD, 95%CI)	2.0 (1.1) (1.6; 2.5)	2.4 (1.0) (1.8; 3.0)	1.7 (1.0) (1.0; 2.3)	0.039
Single organ *n* (%)	8 (33.3)	2 (16.7)	6 (50.0)	0.149
Multiorgan (≥ 2) *n* (%)	16 (66.7)	10 (83.3)	6 (50.0)	0.083
Allergy *n* (%)	5 (20.8)	2 (16.7)	3 (25.0)	> 0.999

*HPF* high power field, *ns* not significant

^#^Data available for 12/12 male and 11/12 female patients

^∞^Data available for 10/12 male and 11/12 female patients

Elevated serum IgG4 was detected in 58.3%, with 25% exceeding five times the upper limit of normal (Table S2). Markedly elevated IgG4 was more common in males (33.3% vs. 16.7%, *p* = 0.036), while normal IgG4 was more frequent in females (66.7% vs. 16.7%, *p* = 0.036; Table S2). Hypocomplementemia occurred in 30%, slightly more in males. Other known relapse risk factors such as elevated total IgG (25%) and eosinophilia (13%) were uncommon. Half had elevated IgE. The IgG4/total IgG ratio was significantly higher in males (0.8, 95%CI 0.2; 3.4 vs. 0.1, 95%CI 0.0; 0.5, *p* = 0.017).

## **Therapeutic course**

Immunosuppressive therapy was administered to 87.5% (Table S3), all receiving glucocorticoids (GC) with a median starting dose of 40 mg/day (95%CI 30.0–60.0). Males received significantly higher initial GC doses (60 mg/day 95%CI 40.0; 80.0 vs. 40 mg/day 95%CI 5.0; 60.0 in females, *p* = 0.041). By last follow-up, 61.9% of treated patients were GC-free, and those still receiving GC had a median dose of 17.5 mg/day (95%CI 5.0; 60.0). Two patients remained on GC monotherapy throughout—one with head and neck involvement, the other with multiorgan disease.

**Fig. 2:**
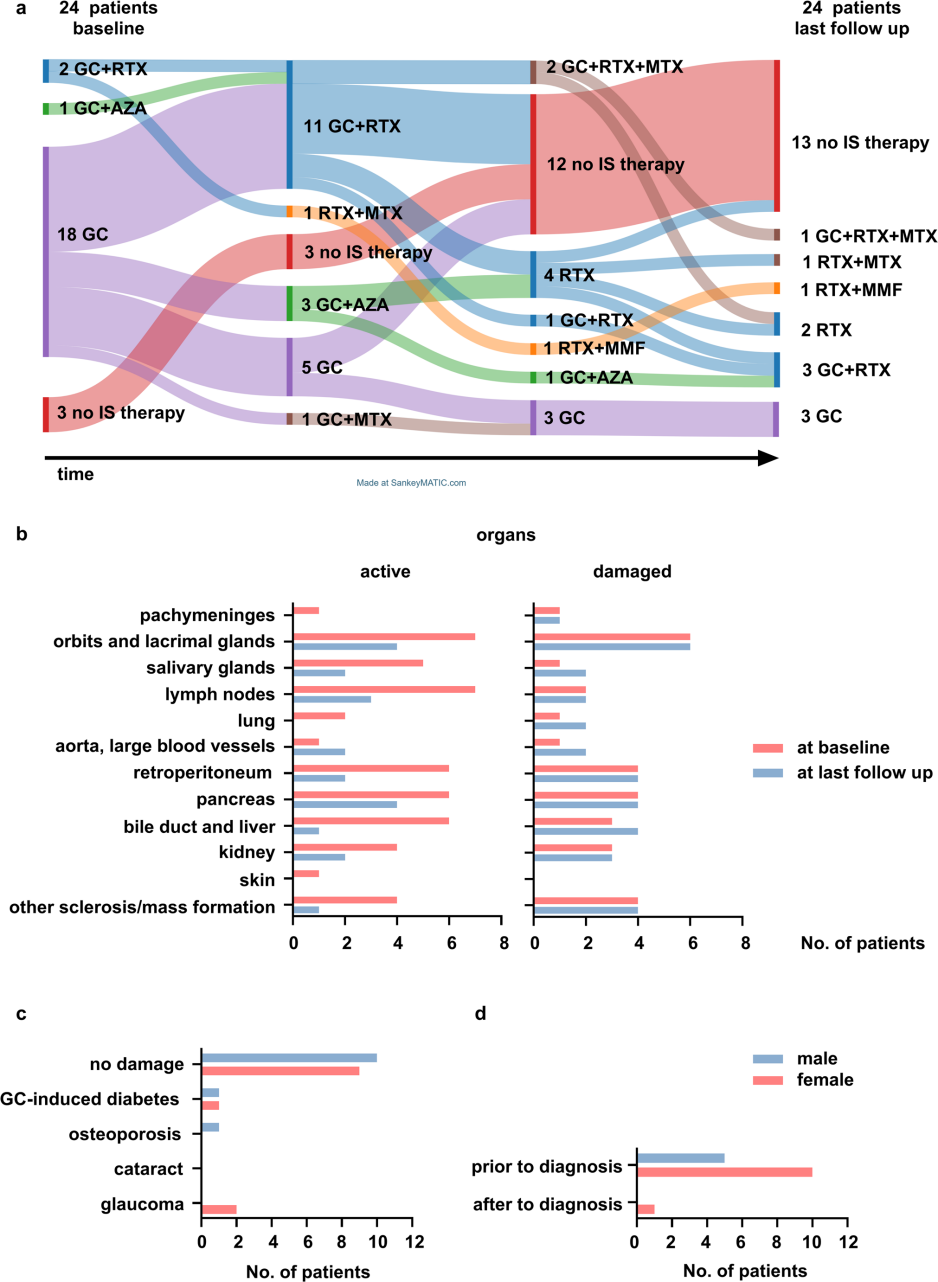
Therapeutic course, outcome, and damage. **a** Flow diagram of therapeutic course from baseline till last follow-up shows great variability **b** Despite heterogenous strategies therapy was effective in all patients regarding active organs (left), and damage only progressed in 16.7% of patients (right). **c** Only 23.8% of patients had any type of Glucocorticoid-induced damage. **d** Surgery was performed in 15/24 patients before diagnosis. *GC* Glucocorticoid, *AZA* Azathioprine, *RTX* Rituximab, *IS* immunosuppressive, *MTX* Methotrexate, *MMF* Mycophenolate Mofetil

Most patients (42.8%) received a single DMARD; only one tried three sequential DMARDs. Rituximab was the predominant DMARD (71.4%), used as first-line DMARD after GC in 57.1% of treated patients, though only two (9.5%) received it upfront combined with GC (Fig. 2a and Table S3). Other DMARDs included azathioprine and methotrexate (four patients each), and mycophenolate mofetil (one patient). By last follow-up, 13 of 21 treated patients were off all immunosuppression; none remained on azathioprine (Fig. 2a). There were no significant differences between male and female patients.

**Fig. 3:**
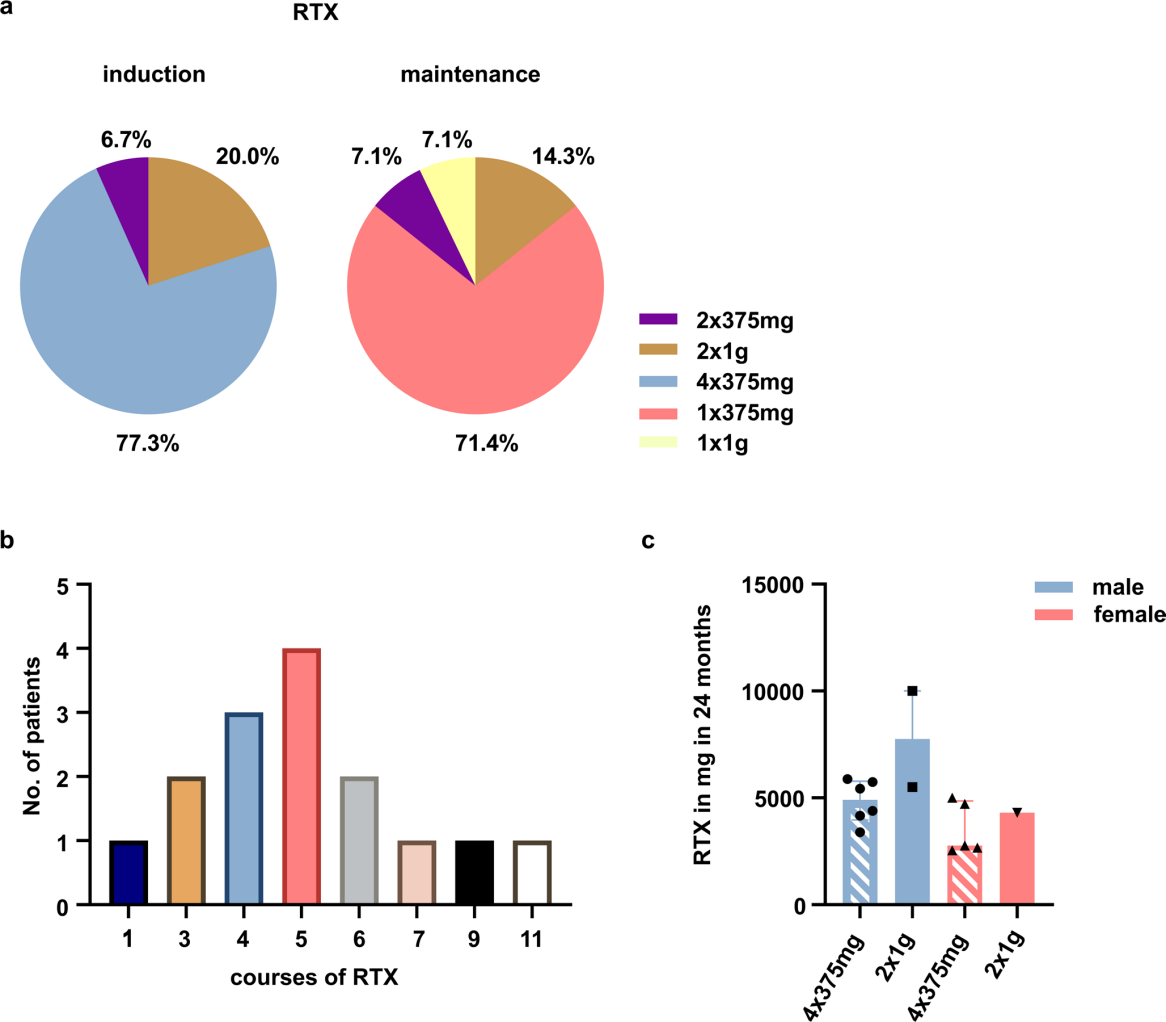
Parameters of Rituximab therapy. **a** Induction therapy with RTX was performed with 4 × 375 mg per m^2^ body surface area and maintenance treatment with 1 × 375 mg per m^2^ body surface area in almost ¾ of cases. **b** Most patients received 4 or 5 courses of RTX. **c** Median doses of RTX within the first 24 months did not differ significantly between the two most common regimens (median and 95%CI). *RTX* Rituximab

The common rituximab induction regimen was 4 × 375 mg/m² (77.3%), followed by 2 × 1 g (20%). Maintenance was mostly 1 × 375 mg/m² (71.4%) or 2 × 1 g (14.3%), with other doses also used (Fig. 3a). B cell depletion was achieved in all tested patients (data missing for 3 of 15 patients). Rituximab was readministered systematically in 85.7% and most received 4–5 courses of rituximab (Table S4 and Fig. 3b). Median cumulative rituximab doses over two years did not differ significantly between regimens (Fig. 3c). The shortest and longest intervals between courses were 6 months (95%CI 5.0; 7.0) and 10 months (95%CI 7.0; 13.0; Table S3). Two females paused rituximab for 33 and 37 months, relapsed, and responded well to rituximab reinduction with 2 × 1 g. No sex differences regarding rituximab were evident. Rituximab treated patients had higher GC doses at last follow-up compared to patients on alternative immunosuppressive agents (20.0 vs. 5.0 mg, 95%CI 10.0; 60.0 and 5.0; 30.0, *p* = 0.232).

### Outcomes and damage

Overall, active organ involvement markedly decreased by the last follow-up, regardless of therapy. Only 4/24 (16.7%) accumulated organ damage during observation, affecting salivary glands, lung, aorta, bile duct, or liver (Fig. 2b). A clinical response was observed in all patients treated with rituximab. A serological response, defined as a reduction in serum IgG4 levels by more than 50% after 3 months, was seen in 42.8% of patients, and a radiological response in 71.4% (pre- and post-treatment imaging was available for 7 of 15 rituximab patients; Table S4). Rituximab patients more frequently had storiform fibrosis on histology (80 vs. 16.7%, *p* = 0.014) and a definite diagnosis by the 2020 RCD criteria (73.3 vs. 16.7%, *p* = 0.020, Table S5). Serum or tissue IgG4 did not differ.

GC-induced damage occurred in 5/21 (20.8%) treated patients, with no sex difference (Fig. 2c). Glaucoma was the most common GC-related complication with 2 cases.

Surgery preceded IgG4-RD diagnosis in 16 patients (62.5%), with only one needing further surgery after diagnosis (Fig. 2d).

One patient died from infectious complications shortly after rituximab induction (toxic megacolon and septicemia). Infusion reactions occurred in two patients (13.3%) corresponding to 2 reactions out of 128 infusions, and did not lead to treatment discontinuation (Table S6). The incidence of infectious complications with rituximab was not significantly higher than that observed with other immunosuppressive therapies. No severe cases of rituximab-induced hypogammaglobulinemia or neutropenia were reported during the long-term follow-up period of a median of 51 months (95%CI 27.0–63.0) after initiation of rituximab (Tables S4 and S6).

Relapses were frequent, affecting 81% of treated patients, without sex difference (Table S3). Nearly half (47.6%) experienced one relapse, 14.3% two, and 19.1% three relapses. Median overall relapse-free survival by Kaplan-Meier curve was 23 months (95%CI 8.0–32.0). About 50% of relapses happened within the first 12 months (Fig S2). Relapses were more frequent after therapy discontinuation (25%) or during GC monotherapy (42.8%). Patients with relapses were more likely to have multiorgan disease (82.3 vs. 28.6%, *p* = 0.011) and more affected organs (2.3 vs. 1.4, 95%CI 1.8; 2.9 and 0.7; 2.1; *p* = 0.028, TableS7). A definite diagnosis of IgG4-RD and higher levels of serum IgG4 were more common with relapse, yet not significant. Other baseline serologic markers did not differ (Table S8). Only patients with a relapse received more than one immunosuppressive therapy over follow-up (1.5 vs. 1.0 95%CI 1.2; 1.9; Table S9). Median GC dose at relapse was 5.0 mg/day (95%CI 5.0; 5.0), with no sex difference (Table S3). In rituximab treated patients relapse rate dropped from 86.7 to 26.7% (Table S4). However, time to relapse on rituximab was shorter with 5.0 (95%CI 3.0; 7.0) versus 10.0 (95%CI 4.0; 20.0) months. All relapse episodes responded well to therapy escalation.

Two patients (one male, one female) developed non–small cell lung cancer at a median of 128.5 months (95%CI 88.0–169.0) after the diagnosis of IgG4-RD (Table S10). In one of these patients, the lung had been previously affected by IgG4-RD.

## Discussion

This study provides valuable insights into induction and long-term maintenance strategies for IgG4-RD, highlighting the efficacy and safety of long-term rituximab as well as the need for further research and standardization.

High relapse rates are well-documented in IgG4-RD, with up to 50% relapsing after discontinuing DMARDs and GC, even after quiescent disease ≥ 12 months [13]. Continuing a DMARD alone is as effective as combining it with GC (relapse rate 14.2 vs. 12.2%). Our findings align, with two-thirds achieving GC-free remission at last follow-up. Although not prospectively studied, GC-free maintenance should be the goal after the first year if disease is inactive and relapse risk low, to avoid GC-induced damage. Long-term GC use doubles cardiovascular risk even below 5 mg/day, and diabetes, hypertension, and osteoporosis are more frequent in IgG4-RD [14, 15]. Higher GC doses in males in this study likely reflect weight-based dosing. Future studies should determine the lowest effective GC induction dose, with or without DMARDs. Evidence suggests 0.5 mg/kg GC is as effective as 1.0 mg/kg, but efficacy of lower doses and shorter treatment periods - especially combined with B cell depletion - remains unproven [16]. A 24-month fixed rituximab regimen administered upon B cell recurrence limits cumulative GC exposure, with 77% relapse-free for an additional year, supporting rituximab maintenance to minimize GC toxicity [17]. IgG4-RD patients also face an increased mortality risk (HR 2.51 vs. general population), further highlighting the need for GC reducing strategies and of cardiovascular and metabolic monitoring, as in other rheumatic diseases [18].

The optimal DMARD for inducing and maintaining remission in IgG4-RD is not established; however, B cell–targeting therapy is emerging as a first-line option. Inebilizumab (anti-CD19) improved flare-free remission (57.4% of treated patients) in a Phase III trial at 12 months and has been approved for therapy by regulatory authorities in April 2025 in the United States [10]. Selective inhibition of Bruton’s tyrosine kinase with rilzabrutinib led to flare-free remission in 70% at week 52 without additional treatment in a Phase II trial [19]. A meta-analysis of rituximab (374 patients, mean follow-up 23.4 ± 16.3 months) showed a 97.3% induction response rate and substantially lower relapse rates during maintenance (2.8% vs. 21.5%) [20]. Rituximab efficacy was confirmed in a prospective cohort of 115 European patients, with about 70% responding after six months regardless of phenotype [21]. However, follow-up was limited to 12 months, and the rituximab regimen was fixed at 2 × 1 g induction, with 62.6% receiving a second dose at 6 months. Thalidomide reduced relapses (13.8% vs. 67.8% placebo) in a 12-month multicenter RCT, but 77.5% of adverse events occurred in thalidomide-treated patients, and teratogenicity restricts its use to rescue therapy [22]. A Cochrane review evaluating immunosuppressant benefits and harms is ongoing and may guide future trials [23].

Our data confirm that rituximab induction regimen (4 × 375 mg/m² vs. 2 × 1 g) does not affect remission rates. Maintenance with as little as 375 mg/m² is feasible, potentially reducing costs and side effects. The high rituximab use in our cohort (71.4% vs. 14.0% in US claims data) reflects tertiary center practice in Germany, where off-label rituximab is often reimbursed and disease burden high [18]. Nevertheless, severe adverse effects were rare and further support the use of rituximab as maintenance therapy [24]. International guidelines for B cell depletion therapy would improve access. Approval of inebilizumab on the other hand may increase costs and limit off-label rituximab use despite proven efficacy. The observed high relapse rate (81%) in this cohort—mainly after therapy cessation, during GC monotherapy or prior to rituximab induction—may be due to long follow-up (median ~ 4.5 years). Relapse rates reach 52% after 18 months of immunosuppressant withdrawal and 41.9% at 19 months post-rituximab [13, 25]. Rituximab interruptions or prolongation of application intervals during the COVID-19 pandemic (56% of patients) may have contributed to this high relapse rate and reduced use at last follow-up. This can possible also explain the relatively high median dose of GC at LFU (17.5 mg/day, 95%CI 5.0; 60.0). Relapse rates may also differ among disease phenotypes [21].

Despite diagnostic advances, median diagnostic delay remains high (10.5 months), delaying therapy and risking cumulative damage. However, this delay is shorter than in prior national (2015: 60 months) and international (2019: 21.6 months) cohorts, reflecting growing awareness [2, 5]. The low rate of elevated serum IgG4 in females (33.3%) highlights limited screening sensitivity and the need for broader diagnostic approaches to avoid sex-specific delays. The high rate of pre-diagnostic surgery (62.5%) underscores the need for better imaging and biomarkers to prevent unnecessary surgery and damage. Combined imaging ([18 F]-FDG and [68Ga]-FAPI PET/CT or MRI) at different timepoints may differentiate malignant from fibroinflammatory lesions and refine disease subtypes, phenotypes and management by detecting additional affected organs—mainly fibroblast-active regions less responsive to immunosuppressive therapy [26–28]. Targeting activated fibroblasts in IgG4-RD is actively researched and may additionally improve outcomes. This is of special interest for disease phenotypes like “Aorta and Retroperitoneum” (A/R) and “Head and Neck-limited” (H/N-L), which show lower remission rates and are more likely to flare under rituximab therapy respectively [21].

The fact that only 50% of patients in this well-characterized tertiary referral center cohort were classified as having “definite” IgG4-RD according to the 2020 RCD criteria highlights the reduced sensitivity of these criteria for specific disease phenotypes, particularly the A/R phenotype. In a comparative analysis of the 2020 RCD and 2019 ACR/EULAR classification criteria across IgG4-RD phenotypes, only 2 of 18 A/R patients were diagnosed as definite IgG4-RD by the 2020 RCD criteria, and only 5 met the 2019 ACR/EULAR criteria [29]. Given this, and considering the long follow-up period of 4.5 years that allowed for re-evaluation and reclassification, we believe our results accurately represent clinical practice.

Our cohort did not show male predominance, but males had higher baseline IgG4 levels and more multiorgan involvement [6]. This might partially be explained by usage of the 2019 ACR/EULAR classification criteria in most of other studies. However, a US claims-based analysis also indicated weaker male predominance, with 56.7% females [18]. Pancreatic and renal disease were more common in males, though not statistically significant due to small sample size. Further studies should clarify sex differences considering subtypes, outcome, and treatment response and keep in mind the low sensitivity of the 2019 ACR/EULAR classification criteria in female patients [6].

Increased malignancy risk in IgG4-RD has been reported repeatedly in different ethnicities and is noted here as well, emphasizing the need for standardized screening algorithms [15, 30, 31]. Two malignancies arising after IgG4-RD diagnosis in this cohort were lung cancers, prompting discussion about regular chest X-rays alongside standard screening. However, only one patient had the previously identified risk factors for malignancy, multiorgan disease and high levels of serum IgG4. Larger epidemiologic studies are needed to identify additional risk factors.

Limitations of this study include single-center, retrospective design, potential selection and information bias, and small sample size. To overcome these limitations, future studies evaluating therapeutic strategies and outcomes, identifying new biomarkers, assessing cardiovascular and malignancy risk and disease phenotypes should be based on multinational registries with standardized protocols. Findings from these studies should be followed up by confirmatory prospective studies. One strength of this study is a well described cohort of IgG4-RD patients cared for at a tertiary referral center by an interdisciplinary team lead by rheumatologists with complete follow-up. Another strength is the long observational period of a median of 54.5 months, which allows for a robust evaluation of therapeutic outcomes and ensures that none of the patients included were misdiagnosed as IgG4-RD, as regular re-evaluation of diagnosis was performed. One particular strength of our study is that it reports aspects of efficacy and safety of maintenance treatment with rituximab in 15 patients over a median follow-up of 51.0 months post rituximab initiation for the first time.

## Conclusion

This single-center, retrospective cohort study of 24 well characterized IgG4-RD patients provides important preliminary data on efficacy and safety of long-term (median follow-up post rituximab initiation 51.0 months) maintenance with rituximab. It supports the current clinical use of rituximab for induction and maintenance treatment regardless of lacking randomized controlled trials, and at the same time highlights the need for further research and standardizations of treatment strategies, particularly regarding disease phenotypes (inflammatory vs. fibrotic), subgroups, and sex differences.

## Supplementary Information

Below is the link to the electronic supplementary material.

Supplementary Material 1

## Data Availability

Data will be made available upon reasonable request to the corresponding author.

## Declarations

### Accountability statement

All authors agree to be accountable for all aspects of the work, ensuring that any questions related to the accuracy or integrity of any part of the work are appropriately investigated and resolved.

### Related congress abstract

Part of the content presented in this article was previously shared in the form of an abstract and poster presentation at the 52nd annual meeting of the German Society of Rheumatology 18th-21st of September 2024 in Duesseldorf, Germany (IgG4-RD - Retrospective cohort study of 25 patients from a tertiary center. VS.06; 10.3205/24dgrh232). The current manuscript provides an extended and detailed report of the study’s methodology, data, and results.
