# Supplementary material for: Long-term treatment patterns and outcomes in IgG4-related disease – a retrospective single-center cohort study focusing on rituximab
Source: Rheumatol Int. 2026 Jan 13;46(1):33. doi: 10.1007/s00296-025-06065-1 (PMC12799707; doi:10.1007/s00296-025-06065-1)
Supplement: Supplementary file 1 — Supplementary Material 1 [file 296_2025_6065_MOESM1_ESM.docx]

**Supplementary Material for**

**Long-term treatment patterns and outcomes in IgG4-related disease – a retrospective single-center cohort study focusing on rituximab**

Ariane Hammitzsch, 0000-0003-2658-4764

Noemi Ferraro

Quirin Bachmann, 0009-0003-0312-9141

Uwe Heemann

Philipp Moog, 0000-0001-5002-8149

**in Rheumatology International**

Table S1 Detailed organ involvement

Table S2 Baseline serologic markers

Table S3 Therapy and Relapse

Table S4 Efficacy of long-term Rituximab

Table S5 Baseline demographic characteristics RTX vs other IS

Table S6 Safety of long-term immunosuppression

Table S7 Baseline demographic characteristics by relapse status

Table S8 Baseline serologic markers by relapse status

Table S9 Therapy by relapse

Table S10 Malignancy details overall and by sex

Figure S1 Flow chart of study showing inclusion and exclusion criteria

Fig S2 Kaplan-Meier plot of the risk of disease relapse following rituximab in 15 IgG4-RD patients

STROBE checklist

| **Table S1 Detailed organ involvement at baseline overall and by sex** | | | | |
| --- | --- | --- | --- | --- |
|  | All (n=24) | Male (n=12) | Female (n=12) | p value |
| Number of organs affected at baseline (mean, SD) | 2.0 (1.1) | 2.4 (1.0) | 1.6 (1.0) | 0.039 |
| Pachymeninges | 1 (4.2) | 0 (0.0) | 1 (8.3) | >0.999 |
| Head and Neck n (%) | 10 (41.7) | 6 (50.0) | 4 (33.3) | 0.680 |
| Pancreato-Hepatobiliary n (%) | 7 (29.2) | 4 (33.3) | 3 (25.0) | >0.999 |
| Lymph nodes n (%) | 7 (29.2) | 3 (25.0) | 4 (33.3) | >0.999 |
| Lung n (%) | 2 (8.3) | 1 (8.3) | 1 (8.3) | >0.999 |
| Aorta, large blood vessels n (%) | 1 (4.2) | 1 (8.3) | 0 (0.0) | >0.999 |
| Retroperitoneum, n (%) | 6 (25.0) | 4 (33.3) | 2 (16.7) | 0.640 |
| Kidney n (%) | 4 (16.7) | 4 (33.3) | 0 (0.0) | 0.093 |
| Skin n (%) | 1 (4.2) | 0 (0.0) | 1 (8.3) | >0.999 |
| Other sclerosis/mass n (%) | 4 (16.7) | 2 (16.7) | 2 (16.7) | >0.999 |

| **Table S2 Baseline serological markers overall and by sex** | | | | |  |
| --- | --- | --- | --- | --- | --- |
|  | All (n=24) | Male (n=12) | Female (n=12) | p value |  |
| IgG4 concentration | |  |  |  |  |
| Normal n (%) | | 10 (41.7) | 2 (16.7) | 8 (66.7) | 0.036 |
| >normal<2xULN n (%) | | 4 (16.7) | 3 (25.0) | 1 (8.3) | 0.590 |
| >2xULN <5xULN n (%) | | 4 (16.7) | 3 (25.0) | 1 (8.3) | 0.590 |
| >5xULN n (%) | | 6 (25.0) | 4 (33.3) | 2 (16.7) | 0.036 |
| C3 and/or C4 <LLN n (%)^#^ | 6 (30.0) | 5 (50.0) | 1 (10.0) | 0.141 |  |
| Eosinophils (%, median, 95%CI)^∞^ | 4.0 (1.0; 4.0) | 4.0 (1.0; 5.0) | 2.0 (1.0; 5.0) | 0.365 |  |
| Eosinophils (cells/µl, median, 95%CI)^∞^ | 248 (115.0; 333.0) | 306 (107.0; 413.0) | 191 (83.0; 430.0) | 0.316 |  |
| Eosinophils >500/µl n (%)^∞^ | 3 (13.0) | 2 (16.7) | 1 (9.1) | >0.999 |  |
| Eosinophils >4% n (%)^∞^ | 6 (26.1) | 4 (33.3) | 2 (18.2) | 0.640 |  |
| IgE (KU/L, median, 95%CI)^*^ | 93.0 (25.0; 139.0) | 82.0 (25.0; 819.0) | 104.0 (10.0; 318.0) | 0.518 |  |
| IgE >ULN n (%)^*^ | 11 (50.0) | 5 (45.5) | 6 (54.5) | >0.999 |  |
| CRP (mg/dl, median, 95%CI) | 0.2 (0.1; 0.7) | 0.2 (0.1; 0.7) | 0.2 (0.1; 1.3) | 0.913 |  |
| IgG (mg/dl, median, 95%CI) | 1018.0 (750.0; 1203.0) | 845.5 (388.0; 1919.0) | 1059.0 (880.0; 1203.0) | 0.712 |  |
| IgG >ULN n (%) | 6 (25.0) | 4 (33.3) | 2 (16.7) | 0.640 |  |
| IgG4/IgG (median, 95%CI) | 0.4 (0.1; 0.9) | 0.8 (0.2; 3.4) | 0.1 (0.0; 0.5) | 0.017 |  |

ULN, upper limit of normal; LLN, lower limit of normal.

^#^ data available for 10/12 male and 10/12 female patients.

^∞^ data available for 12/12 male and 11/12 female patients.

^*^ data available for 10/12 male and 10/12 female patients.

| **Table S3 Therapy and relapse** | | | | |
| --- | --- | --- | --- | --- |
|  | All (n=24) | Male (n=12) | Female (n=12) | p value |
| Immunosuppression ever n (%) | 21 (87.5) | 12 (100.0) | 9 (75.0) | 0.217 |
| GC n (%) | 21 (100.0) | 12 (100.0) | 9 (100.0) | >0.999 |
| RTX n (%) | 15 (71.4) | 9 (75.0) | 6 (66.7) | >0.999 |
| AZA n (%) | 4 (19.0) | 2 (16.6) | 2 (22.2) | >0.999 |
| MTX n (%) | 4 (19.0) | 1 (8.3) | 3 (33.3) | 0.272 |
| MMF n (%) | 1 (4.8) | 1 (8.3) | 0 (0.0) | >0.999 |
| BL dose of GC (mg/d, median, 95%CI) | 40.0 (30.0; 60.0) | 60 (40.0; 80.0) | 40.0 (5.0; 60.0) | 0.041 |
| Patients free of GC at LFU n (%) | 13 (61.9) | 7 (58.3) | 6 (66.7) | >0.999 |
| LFU dose of GC (mg/dl, median, 95%CI) | 17.5 (5.0; 60.0) | 30.0 (5.0; 60.0) | 10.0 (5.0; 15.0) | 0.161 |
| Total number of therapies other than GC n (mean, SD) | 1.5 (0.6) | 1.5 (0.7) | 1.4 (0.5) | 0.835 |
| =1 | 9 (42.8) | 5 (41.2) | 4 (44.4) | >0.999 |
| =2 | 6 (28.6) | 3 (25.0) | 3 (33.3) | >0.999 |
| =3 | 1 (4.8) | 1 (8.3) | 0 (0.0) | >0.999 |
| Treatment courses of RTX (n, median, 95%CI) | 5.0 (4.0; 7.0) | 5.0 (3.0; 8.0) | 4.5 (4.0; 9.0) | 0.707 |
| Interval of RTX (months, median, 95%CI) | 7.0 (7.0; 7.0) | 7.0 (6.0; 7.0) | 7.0 (7.0; 9.0) | 0.216 |
| shortest interval (months, median, 95%CI) | 6.0 (5.0; 7.0) | 6.0 (5.0; 7.0) | 5.0 (3.0; 7.0) | 0.133 |
| longest interval (months, median, 95%CI) | 10.0 (7.0; 13.0) | 8.5 (7.0; 16.0) | 10.0 (7.0; 13.0) | >0.999 |
| Relapse ever n (%) | 17 (81.0) | 10 (83.3) | 7 (77.8) | >0.999 |
| =1 n (%) | 10 (47.6) | 8 (66.7) | 2 (22.2) | 0.080 |
| =2 n (%) | 3 (14.3) | 1 (8.3) | 2 (22.2) | 0.553 |
| =3 n (%) | 4 (19.0) | 1 (8.3) | 3 (33.3) | 0.272 |
| Pausing of RTX due to Corona pandemic, no. (%) | 7 (58.3) | 4 (66.7) | 3 (50.0) | >0.999 |
| Relapse on n (% of total episodes) |  |  |  |  |
| no therapy | 7 (25.0) | 2 (15.4) | 5 (33.3) | 0.395 |
| GC | 12 (42.8) | 7 (53.8) | 5 (33.3) | 0.445 |
| RTX | 4 (14.3) | 2 (15.4) | 2 (13.3) | >0.999 |
| AZA | 4 (14.3) | 1 (7.7) | 3 (20.0) | 0.600 |
| MTX | 1 (3.6) | 1 (7.7) | 0 (0.0) | 0.464 |
| GC dose at relapse (mg/dl, median, 95%CI)^#^ | 5.0 (5.0; 5.0) | 5.0 (1.2; 7.5) | 5.0 (5.0; 10.0) | 0.483 |

GC, Glucocorticoid; RTX, Rituximab; AZA, Azathioprine; MTX, Methotrexate; MMF, Mycophenolate mofetil; BL, baseline; LFU, last follow up.

^#^ data available for 7 of 11 episodes.

| **Table S4 Efficacy of long-term treatment with Rituximab** | | | | | |
| --- | --- | --- | --- | --- | --- |
|  |  | | RTX (n=15)^#^ |  |  |
| Duration of follow-up (months; median, 95%CI) | |  | 53.0 (35.0; 67.0) |  |  |
| After first RTX^∞^ | |  | 51.0 (27.0; 63.0) |  |  |
| After last RTX^∞^ | |  | 12.0 (1.0; 19.0) |  |  |
| Treatment before RTX | |  |  |  |  |
| GC n (%) | |  | 15 (100.0) |  |  |
| AZA n (%) | |  | 4 (26.7) |  |  |
| MTX n (%) | |  | 0 (0.0) |  |  |
| MMF n (%) | |  | 0 (0.0) |  |  |
| Treatment type RTX^∞^ | |  |  |  |  |
| Systematic/maintenance treatment n (%) | |  | 12 (85.7) |  |  |
| Retreatment for relapse n (%) | |  | 2 (14.3) |  |  |
| DMARD treatment concurrent with RTX n (%)^*^ | |  | 4 (26.7) |  |  |
| Patients free of GC at LFU n (%) | |  | 10 (66.7) |  |  |
| LFU dose of GC (mg/dl, median, 95%CI) | |  | 20.0 (10.0; 60.0) |  |  |
| Serological response | |  |  |  |  |
| Serum IgG4 decrease >50% within 3 months post RTX induction n (%)^§^ | |  | 6 (42.8) |  |  |
| Serum IgG4 normalisation ever post RTX induction (<135 mg/dl) n (%) | |  | 7 (63.6) |  |  |
| Overall clinical response | |  | 15 (100.0) |  |  |
| Radiological response n (%)^±^ | |  | 5 (71.4) |  |  |
| Metabolic response (FDG-PET/CT or MRI) n (%)^©^ | |  | 1 (100.0) |  |  |
| Relapse ever prior to RTX n (%) | |  | 13 (86.7) |  |  |
| = 1 n (%) | |  | 8 (61.5) |  |  |
| = 2 n (%) | |  | 4 (30.8) |  |  |
| = 3 n (%) | |  | 1 (7.7) |  |  |
| Time to relapse from last induction months (median, 95%CI) | |  | 10.0 (4.0; 20.0) |  |  |
| Relapse ever while on RTX n (%) | |  | 4 (26.7) |  |  |
| =1 n (%) | |  | 3 (20.0) |  |  |
| =2 n (%) | |  | 1 (6.7) |  |  |
| Time to relapse from last RTX months (median, 95%CI) | |  | 5.0 (3.0; 7.0) |  |  |

RTX, Rituximab; GC, Glucocorticoid; AZA, Azathioprin; MTX, Methotrexate; MMF, Mycophenolate mofetil; LFU, last follow up.

^#^ 21 patients were treated with immunosuppressive therapy in total.

^∞^ one patient had only a single course of RTX.

^*^ 3 patients treated with MTX, one patient with MMF.

^§^ pre RTX IgG4 Serum level not available for 1/15 patients. One additional patient reached a serum IgG4 decrease by >50% at month 6 post RTX induction.

^±^ pre and post RTX imaging only available for 7/15 patients.

^©^ pre and post RTX PET-CT or MRI only available for 1/15 patients.

| **Table S5 Baseline demographic characteristics – Rituximab vs other Immunosuppression** | | | | |  |
| --- | --- | --- | --- | --- | --- |
|  | All (n=21) | RTX (n=15) | Other IS (n=6) | p value |  |
| Age at diagnosis (median, 95%CI) | 52.0 (37.0; 61.0) | 54.0 (37.0; 61.0) | 50.0 (21.0; 70.0) | 0.805 |  |
| Time to diagnosis (months; median, 95%CI) | 11.0 (2.0; 28.0) | 10.0 (1.0; 103.0) | 11.0 (0.0; 197.0) | 0.924 |  |
| Duration of follow-up (months; median, 95%CI) | 56.0 (37.0; 67.0) | 53.0 (35.0; 67.0) | 60.5 (34.0; 97.0) | 0.391 |  |
| Male n (%) | 12 (57.1) | 9 (60.0) | 3 (50.0) | >0.999 |  |
| Race |  |  |  |  |  |
| Caucasian | 20 (95.2) | 14 (93.3) | 6 (100.0) | >0.999 |  |
| Asian | 1 (4.8) | 1 (6.7) | 0 (0.0) | >0.999 |  |
| IgG4 concentration (mg/dL, median, 95%CI) | 393.0 (106.0; 1100.0) | 406.0 (63.2; 1430.0) | 388.5 (52.6; 4000.0) | >0.999 |  |
| Biopsy performed n (%) | 21 (100.0) | 15 (100.0) | 6 (100.0) | >0.999 |  |
| Obliterative phlebitis n (%)^#^ | 3 (14.3) | 2 (13.3) | 1 (16.7) | >0.999 |  |
| Storiform fibrosis n (%)^∞^ | 13 (61.9) | 12 (80.0) | 1 (16.7) | 0.014 |  |
| >10 IgG4+ cells/HPF n (%)^*^ | 16 (76.2) | 12 (80.0) | 4 (66.7) | 0.597 |  |
| 2020 revised comprehensive diagnostic criteria n (%) | |  |  |  |  |
| Definite | | 12 (57.1) | 11 (73.3) | 1 (16.7) | 0.020 |
| Probable | | 7 (33.3) | 4 (26.7) | 3 (50.0) | 0.354 |
| Possible | | 2 (9.5) | 0 (0.0) | 2 (33.3) | 0.071 |
| Number of organs affected at baseline (mean, SD, 95%CI) | 2.2 (1.0) (1.8; 2.7) | 2.3 (1.0) (1.7; 2.8) | 2.2 (1.2) (0.9; 3.4) | 0.851 |  |
| Allergy n (%) | | 5 (20.8) | 4 (31.2) | 1 (0.0) | >0.999 |
| Malignancy n (%) | | 4 (16.6) | 4 (26.7) | 0 (0.0) | 0.281 |

RTX, rituximab; IS, immunosuppression; HPF, high power field.

^#^ data available for 14/15 RTX patients and 6/6 other IS patients.

^∞^ data available for 14/15 RTX patients and 6/6 other IS patients.

^*^ data available for 12/15 RTX patients and 6/6 other IS patients.

| **Table S6 Safety of long-term treatment with Rituximab vs other Immunosuppression** | | | | |
| --- | --- | --- | --- | --- |
|  | All (n=21) | RTX (n=15)^#^ | Other IS (n=6) | p value |
| Infusion reactions n (%)∞ | na | 2.0 (13.3) | na | na |
| per infusions n (%) | na | 2/128 (2.6) | na | na |
| Infections n (%) | 7 (33.3) | 6 (40.0) | 1 (16.7) | 0.613 |
| Airways | 5 | 5 | 0 | 0.262 |
| Skin | 1 | 1 | 0 | >0.999 |
| Urogenital | 2 | 2 | 0 | >0.999 |
| Gastrointestinal | 3 | 2 | 1 | >0.999 |
| Mastoiditis | 1 | 1 | 0 | >0.999 |
| Septic joint | 1 | 0 | 1 | >0.999 |
| IgG < 400 mg/dl n (%) | 0 | 0 | 0 | >0.999 |
| IgG < LLN n (%) | 4 | 4 | 0 | 0.281 |
| Lowest IgG mg/dl (median, 95%CI)^*^ | na | 617.5 (611.0; 672.0) | na | na |
| Neutropenia n (%) | 0 | 0 | 0 | >0.999 |
| Death n (%) | 1 (4.8) | 1 (6.7) | 0 (0.0) | >0.999 |

RTX, rituximab; IS, immunosuppression; na, not applicable; LLN, lower limit of normal.

^#^ 4 patients were treated with RTX in combination with other immunosuppressants over time.

^∞^ one syncope and one chest pain; each a singular episode.

^*^ based on values below the LLN in 4 patients.

| **Table S7 Baseline characteristics by relapse status** | | | | |  |
| --- | --- | --- | --- | --- | --- |
|  | All (n=24) | Relapse (n=17) | Non-Relapse (n=7) | p value |  |
| Age at diagnosis (median, 95%CI) | 52.0 (33.2; 61.0) | 52.0 (37.0; 58.0) | 52.0 (28.0; 73.0) | 0.587 |  |
| Time to diagnosis (months; median, 95%CI) | 10.5 (2.0; 27.2) | 11.0 (2.0; 103.0) | 5.0 (0.0; 28.0) | 0.502 |  |
| Duration of follow-up (months; median, 95%CI) | 54.5 (34.2; 67.0) | 61.0 (37.0; 67.0) | 48.0 (2.0; 68.0) | 0.192 |  |
| Male n (%) | 12 (50.0) | 10 (58.8) | 2 (28.6) | 0.371 |  |
| Race |  |  |  |  |  |
| Caucasian | 22 (91.7) | 16 (94.1) | 6 (85.7) | 0.507 |  |
| Asian | 2 (5.9) | 1 (8.3) | 1 (14.3) | 0.507 |  |
| IgG4 concentration (mg/dL, median, 95%CI) | 361.5 (73.9; 1036) | 406.0 (155.0; 1100.0) | 118 (52.6; 3030.0) | 0.260 |  |
| Biopsy performed n (%) | 24 (100.0) | 17 (100.0) | 7 (100.0) | ns |  |
| Obliterative phlebitis n (%)^#^ | 3 (12.5) | 3 (17.6) | 0 (0.0) | 0.530 |  |
| Storiform fibrosis n (%)^#^ | 15 (62.5) | 11 (64.7) | 4 (57.1) | >0.999 |  |
| >10 IgG4+ cells/HPF n (%)^∞^ | 19 (90.5) | 12 (70.6) | 7 (100.0) | 0.272 |  |
| 2020 revised comprehensive diagnostic criteria n (%) | |  |  |  |  |
| Definite | | 12 (50.0) | 11 (64.7) | 1 (14.3) | 0.069 |
| Probable | | 10 (41.7) | 5 (29.4) | 5 (71.4) | 0.085 |
| Possible | | 2 (8.3) | 1 (5.9) | 1 (14.3) | 0.507 |
| Number of organs affected at baseline (mean, SD, 95%CI) | 2.0 (1.1) (1.6; 2.5) | 2.3 (1.0) (1.8; 2.9) | 1.4 (0.8) (0.7; 2.1) | 0.028 |  |
| Single organ n (%) | 8 (33.3) | 3 (17.6) | 5 (71.4) | 0.011 |  |
| Multiorgan (≥2) n (%) | 16 (66.7) | 14 (82.3) | 2 (28.6) | 0.011 |  |
| Allergy n (%) | | 5 (20.8) | 5 (29.4) | 0 (0.0) | 0.272 |
| Malignancy n (%) | | 4 (16.6) | 4 (23.5) | 0 (0.0) | 0.283 |

HPF, high power field.

^#^ data missing for 1/17 relapse patients and 0/7 non-relapse patients.

^∞^ data available for 14/17 relapse patients and 7/7 non-relapse patients.

| **Table S8 Baseline serological markers overall and by relapse** | | | | |  |
| --- | --- | --- | --- | --- | --- |
|  | All  (n=24) | Relapse  (n=17) | Non-Relapse (n=7) | p value |  |
| IgG4 concentration | |  |  |  |  |
| Normal n (%) | | 10 (41.7) | 5 (29.4) | 5 (71.4) | 0.085 |
| >normal<2xULN n (%) | | 4 (16.7) | 3 (17.6) | 1 (5.9) | >0.999 |
| >2xULN <5xULN n (%) | | 4 (16.7) | 4 (23.5) | 1 (5.9) | >0.999 |
| >5xULN n (%) | | 6 (25.0) | 5 (29.4) | 2 (11.8) | >0.999 |
| C3 and/or C4 <LLN n (%)^#^ | 6 (30.0) | 5 (33.3) | 1 (20.0) | >0.999 |  |
| Eosinophils (%, median, 95%CI)^∞^ | 4.0 (1.0; 4.0) | 4.0 (1.0; 5.0) | 3.0 (1.0; 15.0) | 0.935 |  |
| Eosinophils (cells/µl, median, 95%CI)^∞^ | 248 (115.0; 333.0) | 225.5 (86.0; 357.0) | 277.0 (92.0; 1779.0) | 0.720 |  |
| Eosinophils >500/µl n (%)^∞^ | 3 (13.0) | 2 (12.5) | 1 (5.9) | >0.999 |  |
| Eosinophils >4% n (%)^∞^ | 6 (26.1) | 4 (25.0) | 2 (5.9) | 0.613 |  |
| IgE (KU/L, median, 95%CI)^*^ | 93.0 (25.0; 139.0) | 94.0 (25.0; 318.0) | 71.5 (10.0; 819.0) | 0.747 |  |
| IgE >ULN n (%)^*^ | 11 (50.0) | 8 (50.0) | 3 (50) | >0.999 |  |
| CRP (mg/dl, median, 95%CI) | 0.2 (0.1; 0.7) | 0.2 (0.1; 0.4) | 0.3 (0.1; 2.2) | 0.668 |  |
| IgG (mg/dl, median, 95%CI) | 1018.0 (750.0; 1203.0) | 896.0 (706.0; 1203.0) | 1160.0 (619.9; 3300.0) | 0.260 |  |
| IgG >ULN n (%) | 6 (25.0) | 4 (23.5) | 2 (11.8) | >0.999 |  |
| IgG4/IgG (median, 95%CI) | 0.4 (0.1; 0.9) | 0.8 (0.1; 1.4) | 0.2 (0.0; 0.9) | 0.147 |  |

ULN, upper limit of normal; LLN, lower limit of normal.

^#^ data available for 15/17 relapse and 5/7 non-relapse patients.

^∞^ data available for 16/17 relapse and 7/7 non-relapse patients.

^*^ data available for 16/17 relapse and 6/7 non-relapse patients.

| **Table S9 Therapy by relapse status** | | | | |
| --- | --- | --- | --- | --- |
|  | All (n=24) | Relapse (n=17) | Non-Relapse (n=7) | p value |
| Immunosuppression ever n (%) | 21 (87.5) | 17 (100.0) | 4 (57.1) | 0.017 |
| GC n (%) | 21 (100.0) | 17 (100.0) | 4 (100.0) | >0.999 |
| RTX n (%) | 15 (71.4) | 14 (82.3) | 1 (25.0) | 0.021 |
| AZA n (%) | 4 (19.0) | 4 (23.5) | 0 (0.0) | 0.545 |
| MTX n (%) | 4 (19.0) | 4 (23.5 | 0 (0.0) | 0.545 |
| MMF n (%) | 1 (4.8) | 1 (5.9) | 0 (0.0) | >0.999 |
| BL dose of GC (mg/d, median, 95%CI) | 40.0 (30.0; 60.0) | 40 (30.0; 60.0) | 60.0 (60.0; 60.0) | 0.141 |
| Patients free of GC at LFU n (%) | 13 (61.9) | 10 (58.8) | 3 (75.0) | >0.999 |
| LFU dose of GC (mg/dl, median, 95%CI) | 17.5 (5.0; 60.0) | 15.0 (5.0; 60.0) | 10.0 | ^#^ |
| Total number of therapies other than GC n (mean, SD, 95%CI) | 1.5 (0.6) (1.2; 1.8) | 1.5 (0.6) (1.2; 1.9) | 1.0 (0.0) | ^#^ |
| =1 | 9 (42.8) | 8 (47.1) | 1 (25.0) | 0.603 |
| =2 | 6 (28.6) | 6 (25.0) | 0 (0.0) | 0.281 |
| =3 | 1 (4.8) | 1 (5.9) | 0 (0.0) | >0.999 |
| Treatment courses of RTX (n, median, 95%CI) | 5.0 (4.0; 6.0) | 5.0 (4.0; 7.0) | 1.0 | ^#^ |
| Interval of RTX (months, median, 95%CI) | 7.0 (7.0; 7.0) | 7.0 (7.0; 7.0) | na^∞^ | ^#^ |
| shortest interval (months, median, 95%CI) | 6.0 (5.0; 7.0) | 6.0 (5.0; 7.0) | na^∞^ | ^#^ |
| longest interval (months, median, 95%CI) | 10.0 (7.0; 15.0) | 10.0 (7.0; 15.0) | na^∞^ | ^#^ |

GC, Glucocorticoid; RTX, Rituximab; AZA, Azathioprine; MTX, Methotrexate; MMF, Mycophenolate mofetil; BL, baseline; LFU, last follow up.

^#^ no statistics as only 1 patient in the non-relapse group.

^∞^ the one patient in the non-relapse group had only one course of RTX.

| **Table S10 Malignancy details overall and by sex** | | | | |
| --- | --- | --- | --- | --- |
|  | All (n=24) | Male (n=12) | Female (n=12) | p value |
| Malignancy n (%) | 4 (16.6) | 1 (8.3) | 3 (25.0) | 0.590 |
| Prior to diagnosis n (%) | 1 (4.2) | 0 (0.0) | 1 (8.3)^#^ | >0.999 |
| Time from cancer to diagnosis months (median, 95%CI) | 60 (36.0; 84.0) | 0 (0.0) | 60 (36.0; 84.0) | na |
| Post diagnosis n (%) | 2 (8.4) | 1 (8.3)^∞^ | 1 (8.3)^∞^ | >0.999 |
| Time from diagnosis to cancer months (median, 95%CI) | 128.5 (88.0;169.0) | 88.0 | 169.0 | na |
| Concurrent with diagnosis n (%)^*^ | 1 (4.2) | 0 (0.0) | 1 (8.3) | >0.999 |

^#^ one patient with two malignancies (thyroid and breast) prior to IgG4-RD diagnosis.

^∞^ non-small cell lung cancer.

^*^ neuroendocrine tumour of the ileum.

**
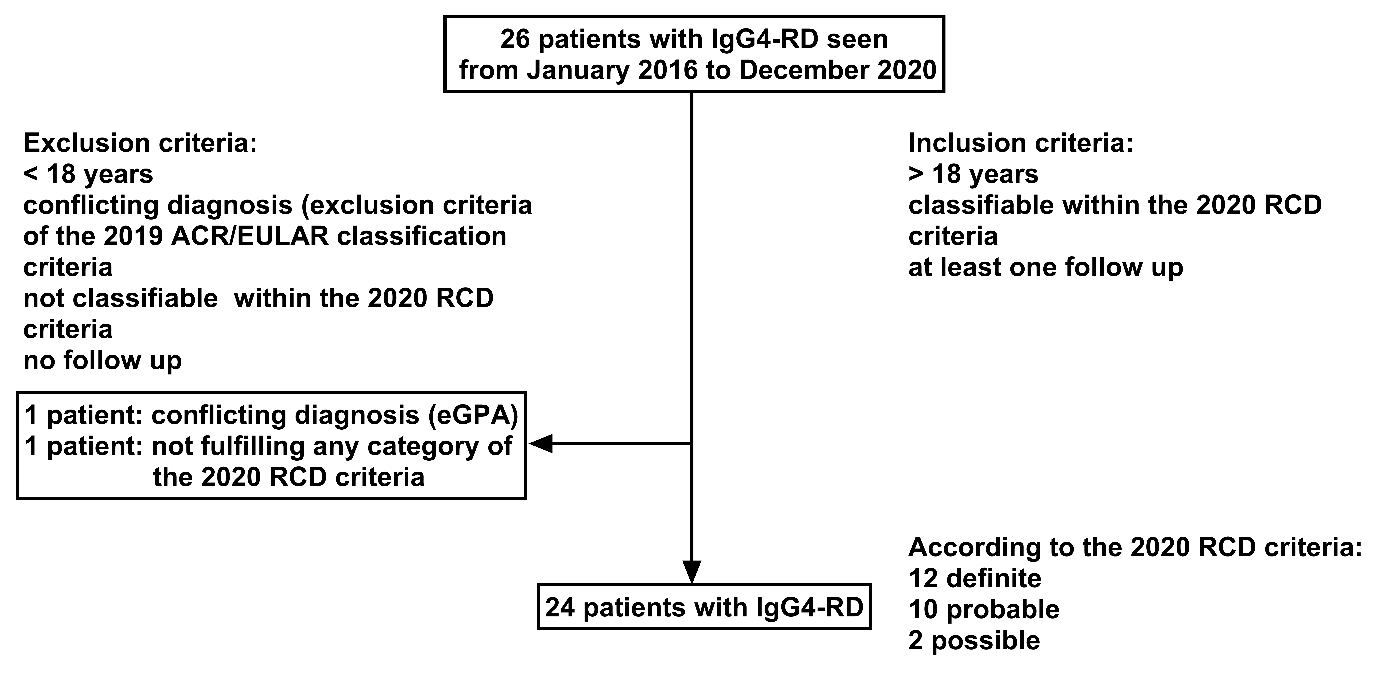
**

**Fig S1 Flow chart of study showing inclusion and exclusion criteria**

**
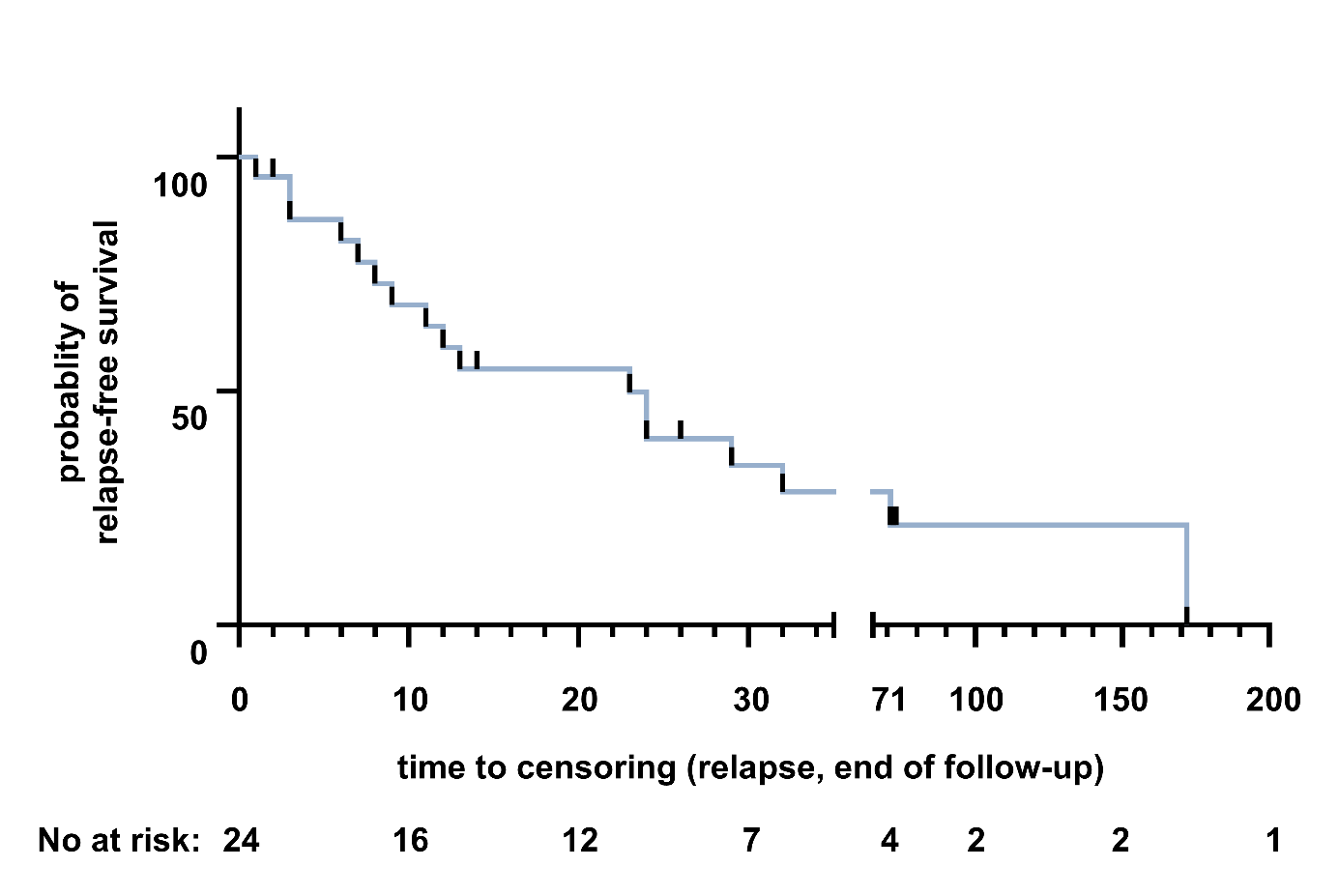
**

**Fig S2 Kaplan-Meier plot of the risk of disease relapse in 24 IgG4-RD patients**

Median relapse-free survival by Kaplan-Meier curve 23 months (95%CI: 8-32). 50% of relapses within the first 12 months. Censored for relapse and end of follow-up. In one patient end of follow-up corresponded to death due to infectious complications.

STROBE Statement—checklist of items that should be included in reports of observational studies

|  | **Item No.** | **Recommendation** | **Page  No.** | **Relevant text from manuscript** |
| --- | --- | --- | --- | --- |
| **Title and abstract** | 1 | (*a*) Indicate the study’s design with a commonly used term in the title or the abstract | Title page (1) | Long-term treatment patterns and outcomes in IgG4-RD – a retrospective single-center cohort study focusing on rituximab |
|  |  | (*b*) Provide in the abstract an informative and balanced summary of what was done and what was found | 2 | Abstract |
| **Introduction** | | | |  |
| Background/rationale | 2 | Explain the scientific background and rationale for the investigation being reported | 3 | Introduction |
| Objectives | 3 | State specific objectives, including any prespecified hypotheses | 3 | “This study retrospectively examines the clinical course, treatments, and outcomes of 24 IgG4-RD patients at a German tertiary center, focusing on therapeutic strategies and disease progression, with particular attention to long-term maintenance with rituximab.” |
| **Methods** | | | |  |
| Study design | 4 | Present key elements of study design early in the paper | 2  3-4  (Supplementary material) 14 | Methods part in Abstract  Methods  Fig S1 – Flow chart of study showing inclusion and exclusion criteria |
| Setting | 5 | Describe the setting, locations, and relevant dates, including periods of recruitment, exposure, follow-up, and data collection | 3-4 | Study population and Data collection in Methods part |
| Participants | 6 | (*a*) *Cohort study*—Give the eligibility criteria, and the sources and methods of selection of participants. Describe methods of follow-up | 3-4  4  (Supplementary material) 14 | Study population  Data collection  Fig S1 – Flow chart of study showing inclusion and exclusion criteria |
|  |  | (*b*) *Cohort study*—For matched studies, give matching criteria and number of exposed and unexposed | na | na |
| Variables | 7 | Clearly define all outcomes, exposures, predictors, potential confounders, and effect modifiers. Give diagnostic criteria, if applicable | 3-4 | Study population (relapse and response, 2020 Revised Comprehensive Diagnostic Criteria for IgG4-RD) |
| Data sources/ measurement | 8* | For each variable of interest, give sources of data and details of methods of assessment (measurement). Describe comparability of assessment methods if there is more than one group | 3-4  4  (Supplementary material) 2-13 | Study population  Data collection  Table 1  Table S1 to S10 |
| Bias | 9 | Describe any efforts to address potential sources of bias | 3-4 (Methods)  5 (Results)  e.g. Table 1 and Table S2  e.g. Outcomes and Damage 6  11 | Clear description of study size and reasons for exclusion of patients  Missing data were clearly indicated in the respective tables and text.  Discussion of study limitations in the Discussion |
| Study size | 10 | Explain how the study size was arrived at | 3-4  (Supplementary material) 14  5 | Methods  Fig S1 – Flow chart of study showing inclusion and exclusion criteria  Results |

Continued on next page

| Quantitative variables | 11 | Explain how quantitative variables were handled in the analyses. If applicable, describe which groupings were chosen and why | 4-5 | Statistical analysis in Methods |
| --- | --- | --- | --- | --- |
| Statistical methods | 12 | (*a*) Describe all statistical methods, including those used to control for confounding | 4-5 | Statistical analysis in Methods |
|  |  | (*b*) Describe any methods used to examine subgroups and interactions | 4-5 | Statistical analysis in Methods |
|  |  | (*c*) Explain how missing data were addressed | e.g. Table 1 and Table S2  e.g. Outcomes and Damage 6 | Missing data were clearly indicated in the respective tables and text. |
|  |  | (*d*) *Cohort study*—If applicable, explain how loss to follow-up was addressed  *Case-control study*—If applicable, explain how matching of cases and controls was addressed  *Cross-sectional study*—If applicable, describe analytical methods taking account of sampling strategy | Fig S2 | Loss to follow-up was censored in Kaplan-Meier analysis and Follow-up time was adjusted |
|  |  | (*e*) Describe any sensitivity analyses | Supplementary Tables S3, S4, S2 and S8 | Results remained similar when groups were changed (male vs. female, relapse vs non-relapse, rituximab vs other immunosuppression)  Mann-Whitney test  Fisher’s exact test |
| **Results** | | | | |
| Participants | 13* | (a) Report numbers of individuals at each stage of study—eg numbers potentially eligible, examined for eligibility, confirmed eligible, included in the study, completing follow-up, and analysed | (Supplementary material) 14 | Fig S1 – Flow chart of study showing inclusion and exclusion criteria  Results  Supplementary material |
|  |  | (b) Give reasons for non-participation at each stage | (Supplementary material) 14 | Fig S1 – Flow chart of study showing inclusion and exclusion criteria  Results  Supplementary material |
|  |  | (c) Consider use of a flow diagram | Supplementary material) 14 | Fig S1 – Flow chart of study showing inclusion and exclusion criteria |
| Descriptive data | 14* | (a) Give characteristics of study participants (eg demographic, clinical, social) and information on exposures and potential confounders |  | Table 1  Table S5  Table S7 |
|  |  | (b) Indicate number of participants with missing data for each variable of interest |  | Table 1  Table S2, S3, S4, S5, S6, S7, S8 |
|  |  | (c) *Cohort study*—Summarise follow-up time (eg, average and total amount) |  | Table 1  Table S4 |
| Outcome data | 15* | *Cohort study*—Report numbers of outcome events or summary measures over time | Supplementary material) 15 | Fig S2 Kaplan-Meier curve of probability of relapse-free survival |
|  |  | *Case-control study—*Report numbers in each exposure category, or summary measures of exposure |  | *na* |
|  |  | *Cross-sectional study—*Report numbers of outcome events or summary measures |  | *na* |
| Main results | 16 | (*a*) Give unadjusted estimates and, if applicable, confounder-adjusted estimates and their precision (eg, 95% confidence interval). Make clear which confounders were adjusted for and why they were included |  | IQR reported |
|  |  | (*b*) Report category boundaries when continuous variables were categorized |  | na |
|  |  | (*c*) If relevant, consider translating estimates of relative risk into absolute risk for a meaningful time period |  | na |

Continued on next page

| Other analyses | 17 | Report other analyses done—eg analyses of subgroups and interactions, and sensitivity analyses |  | Supplementary material Relapse vs non-relapse and Rituximab vs other Immunosuppression |
| --- | --- | --- | --- | --- |
| **Discussion** | | | | |
| Key results | 18 | Summarise key results with reference to study objectives | 8-11  11 | Discussion  Conclusion |
| Limitations | 19 | Discuss limitations of the study, taking into account sources of potential bias or imprecision. Discuss both direction and magnitude of any potential bias | 11 | Discussion |
| Interpretation | 20 | Give a cautious overall interpretation of results considering objectives, limitations, multiplicity of analyses, results from similar studies, and other relevant evidence | 8-11 | Discussion |
| Generalisability | 21 | Discuss the generalisability (external validity) of the study results | 8-11 | Discussion |
| **Other information** | |  | | |
| Funding | 22 | Give the source of funding and the role of the funders for the present study and, if applicable, for the original study on which the present article is based |  | Title page |

*Give information separately for cases and controls in case-control studies and, if applicable, for exposed and unexposed groups in cohort and cross-sectional studies.

**Note:** An Explanation and Elaboration article discusses each checklist item and gives methodological background and published examples of transparent reporting. The STROBE checklist is best used in conjunction with this article (freely available on the Web sites of PLoS Medicine at http://www.plosmedicine.org/, Annals of Internal Medicine at http://www.annals.org/, and Epidemiology at http://www.epidem.com/). Information on the STROBE Initiative is available at www.strobe-statement.org.
